# Hemodialysis catheter-related bacteremia rate is reduced by the use of 0.1% polyhexanide-betaine in a pediatric unit

**DOI:** 10.1177/11297298251369055

**Published:** 2025-08-29

**Authors:** Francisco Antonio Nieto-Vega, Inmaculada Moreno-González, Begoña Rodríguez-Azor, Ruth María González-Ponce, Verónica Dolores Martínez-Rivera, María Dolores Rico-De-Torres, Ana María Reina-González

**Affiliations:** Pediatric Nephrology Unit, Department of Pediatrics, Regional University Hospital of Málaga, Málaga, Spain

**Keywords:** Hemodialysis, tunneled central venous catheter, catheter-related infection, polyhexanide-betaine, pediatrics

## Abstract

**Background:**

Infection is one of the main catheter-related complications in children undergoing hemodialysis. Polyhexanide-betaine (PHMB-B) is a broad-spectrum biocide that is especially useful for removing biofilms and improving wound healing. However, there is no evidence regarding its use in routine hemodialysis exit-site care.

**Methods:**

In January 2019, we implemented a new exit site care protocol using PHMB-B over 2% chlorhexidine. The aim of our study was to evaluate the effect of this new protocol on catheter-related bacteremia (CRB) and exit-site infection (ESI) rates. For this, we conducted a retrospective chart analysis of pediatric patients admitted to our hemodialysis unit with tunneled catheters from January 2017 to December 2020, comparing incidence rates before and after protocol implementation. CRB was defined as the presence of infectious symptoms without apparent sources, with defervescence after antibiotic treatment and/or catheter removal, with or without microbiological confirmation. ESI was defined as the presence of purulent discharge with/without skin erythema ⩽2 cm from the exit site.

**Results:**

Twenty children (50% female) aged 4 months–15 years old were admitted to our unit, for a total of 6177 catheter days. After the implementation of our new protocol, the CRB event rate decreased from 2.64 (10 events) to 0.41/1000 catheter-days (1 event; *p* = 0.041), and the ESI rate decreased from 2.37 (9 events) to 1.25/1000 catheter-days (3 events; ns). No cutaneous adverse reactions or apparent wearing of the catheter material were observed with the use of PHMB-B.

**Conclusions:**

The use of PHMB-B in routine catheter exit site care could be useful in the prevention of catheter-related infectious complications.

## Introduction

Due to their lower complication rate, arteriovenous fistulas should be the vascular access of choice in hemodialysis patients. Nevertheless, tunneled catheters are the preferred vascular access in children worldwide.^1^ Infection is one of the main catheter-related complications, with an incidence rate ranging from 1.1 to 5.5 events/1000 catheter days, depending on the hemodialysis unit.^2–5^ Several clinical practice guidelines gather multiple strategies oriented to prevent infectious complications.^6,7^ Connection and disconnection from the hemodialysis monitor are crucial moments, where preventive measures have a central role. Due to its good skin tolerance and compatibility with catheter materials and rapid and long-lasting effect, aqueous 0.5%–2% chlorhexidine is the exit-site care antiseptic of choice.^6,7^

During the last few years, aqueous 0.1% polyhexanide-betaine (PHMB-B) solution has emerged as a new bactericidal agent. It has shown high bactericidal activity even against multidrug-resistant pathogens,^8^ higher antibiofilm activity and a more lasting effect than 2% chlorhexidine.^9^ This solution, initially used for wound cleansing and disinfection, has also been tried in skin burn care,^10^ diabetic foot care,^11^ and peritoneal catheter exit-site care.^12^ However, there is no evidence regarding its application in hemodialysis tunneled catheter care.

In January 2019, we decided to implement a new care protocol using PHMB-B instead of 2% chlorhexidine in routine catheter exit site care. After 2 years, we decided to evaluate our results over infectious complications and compare them with the previous years using 2% chlorhexidine.

## Materials and methods

### Study population

A retrospective review of medical records was conducted. All patients 0–17 years old admitted to the Pediatric Hemodialysis Unit at Hospital Regional Universitario de Málaga on hemodialysis through a tunneled catheter from January 2017 to December 2020 were assessed for the present study. We excluded patients admitted for less than 14 days, or on hemodialysis through arteriovenous fistula. All clinical data from our cohort were retrieved from their electronic medical charts.

### Catheter insertion

All catheters inserted in our unit were tunneled, one-cuffed silicone catheters, with a size according to the patient’s weight. They were placed under general anesthesia and ultrasound-guided. After placement, permeability was checked with fluoroscopy. No prophylactic antibiotics were administered prior to catheter placement.

### Routine exit-site care and connection to hemodialysis monitor

Prior to January 2019, our protocol consisted of performing three catheter exit-site cares per week on dialysis days. Before the procedure, patients and nurses wore face masks and performed proper hand hygiene. After removing the catheter dressing, nurses cleaned the exit site with a gauze soaked in aqueous 2% chlorhexidine, wiping from the center and moving out. Then, a new clean dressing was placed on. Thereafter, before checking permeability and connecting the patient to the monitor, both catheter branches and hubs were covered with gauze soaked in aqueous 2% chlorhexidine for at least 2 min. This was followed by vigorous scrubbing of the catheter lumens and hubs using gauze pads soaked in the same solution, in accordance with CDC “Scrub the Hub” protocol recommendations.^13^ At the end of each dialysis session, after disconnection from the hemodialysis monitor, both lumens of the catheter were locked with a 3% sodium heparin solution.

In cases of erythema or purulent discharge, culture samples were withdrawn from the exit site, and after disinfection with aqueous 2% chlorhexidine, we applied a 2% mupirocin ointment. In the case of suspected bacteremia, prior to the initiation of systemic antibiotics, blood cultures were withdrawn from both catheter branches and hemodialysis lines.

Starting January 2019, we made these modifications in our protocol:

- We substituted aqueous 2% chlorhexidine for aqueous PHMB-B solution.
- After removing the catheter dressing, we covered the catheter exit site with gauze soaked in PHMB-B for at least 2 min before initiating cleansing.
- In the case of erythema or purulent discharge, after cleaning with aqueous PHMB-B, we substituted the 2% mupirocin ointment for PHMB-B gel.

### Variables

Our primary endpoints were catheter-related bacteremia (CRB) and exit-site infection (ESI) events. We defined CRB as the presence of infectious symptoms (mainly fever and/or hemodynamic instability) in a patient bearing a tunneled central catheter that, after thorough clinical evaluation and diagnostic workup, does not have any other apparent source, and exhibits defervescence after initiating antibiotics and/or removing the vascular access, regardless of microbiological isolation. On the other hand, we defined ESI as the presence of purulent discharge with or without erythema of the skin ⩽2 cm from the exit site, needing topical or systemic antibiotics for at least 1 week until resolution.

Other studied variables were age, sex, renal disease etiology, immunosuppression, number of hemodialysis sessions per week, catheter days, culture results in case of CRB or ESI, catheter removal due to infectious causes, hemodialysis discontinuation due to kidney transplant, transference to peritoneal dialysis or death.

### Statistical analysis

IBM SPSS Statistics version 25 software was used for statistical analysis and generation of figures. Descriptive statistics were used for demographic data as appropriate. CRB and ESI rates were compared using the Wilcoxon signed-rank test. The Kaplan-Meier method of survival analysis was used to generate the probability of CRB or ESI following the initiation of hemodialysis. A *p* value less than 0.05 in two tails was treated as significant in all tests.

## Results

During this period, we admitted 24 patients to our unit. Two patients were on hemodialysis for less than 14 days, and two patients had an arteriovenous fistula and were therefore excluded from our study. Among the 20 patients included in the study, 9 were admitted and discharged before January 2019, and 5 were admitted after January 2019. Finally, the remaining six were admitted before January 2019 and discharged after, switching from one protocol to the other as of January 2019. All patients were outpatients receiving chronic hemodialysis treatment in our unit. Therefore, 15 patients were treated with the chlorhexidine protocol, and 11 were treated with the PHMB-B protocol. No differences between the basal characteristics of the two groups were found (Table 1).

**Table 1.** Baseline characteristics of patients on hemodialysis.

Characteristics	Chlorhexidine (*n* = 15)	PHMB-B (*n* = 11)
Age (years)	8.1	8.7
Male (%)	8 (53.3%)	5 (45.5%)
Immunosuppression (%)	3 (20%)	2 (18.2%)
Mean HD days/week	3.1	3.4
Mean catheter days/patient	252	217
Total number catheter days	3787	2390

PHMB-B: 0.1% polyhexanide-betaine solution; HD: hemodialysis.

Both protocols were equally well tolerated. We did not find any signs of skin irritation or apparent catheter deterioration attributable to any antiseptic.

The use of PHMB-B was associated with a lower CRB rate. During the study period, 10 patients (66.6%) on chlorhexidine and 1 patient (9%) with PHMB-B developed a CRB event (*p* = 0.025). The CRB rate was 2.64/1000 catheter-days on chlorhexidine and 0.41/1000 catheter-days on PHMB-B (*p* = 0.041). No differences associated with age, sex, immunosuppressive status, or weekly hemodialysis sessions were found (Table 2).

**Table 2.** Clinical characteristics of catheter-related bacteremia events.

Characteristics	Chlorhexidine (*n* = 15)	PHMB-B (*n* = 11)	*p* Values
CRB events	10	1	0.025
CVC removal	1	1	ns
CRB rate	2.64	0.41	0.041
Bacterial isolations	*St aureus* (3) *St hominis* (1) *St epidermidis* (1) *Enterobacter cloacae* (1) *Gordonia spp* (1) Negative (3)	*Pseudomonas aeruginosa* (1)	

PHMB-B: 0.1% polyhexanide-betaine solution; CRB events: catheter-related bacteremia events; CVC removal: central venous catheter removal due to catheter-related bacteremia; CRB rate: number of catheter-related bacteremia events per 1000 catheter-days.

On the other hand, we found a lower, albeit not statistically significant, ESI rate with PHMB-B. During the study period, we had nine ESI events among seven patients (46.6%) on chlorhexidine (2.37/1000 catheter-days), and three ESI events among two patients (18.1%) on PHMB-B (1.25/1000 catheter-days; Table 3).

**Table 3.** Clinical characteristics of exit site infection events.

Characteristics	Chlorhexidine (*n* = 15)	PHMB-B (*n* = 11)	*p* Values
ESI events	9	3	ns
CVC removal	0	0	ns
ESI rate	2.37	1.25	ns
Bacterial isolations	*St aureus* (2) *St hominis* (1) *St epidermidis* (1) *Corynebacterium spp* (1) Negative (4)	*St epidermidis* (1) *St hominis* (1) Negative (1)	

PHMB-B: 0.1% polyhexanide-betaine solution; ESI events: exit-site infection events; CVC removal: central venous catheter removal due to exit-site infections; ESI rate: number of exit-site infection events per 1000 catheter-days.

**Figure 1.**
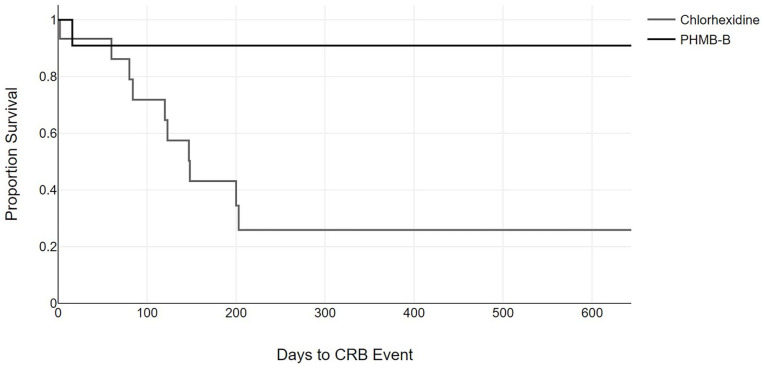
Period of time free of catheter-related bacteremia events. The gray line represents patients who received the chlorhexidine treatment; the black line represents patients who received the polyhexanide treatment (log-rank 4.91; *p* = 0.027).

**Figure 2.**
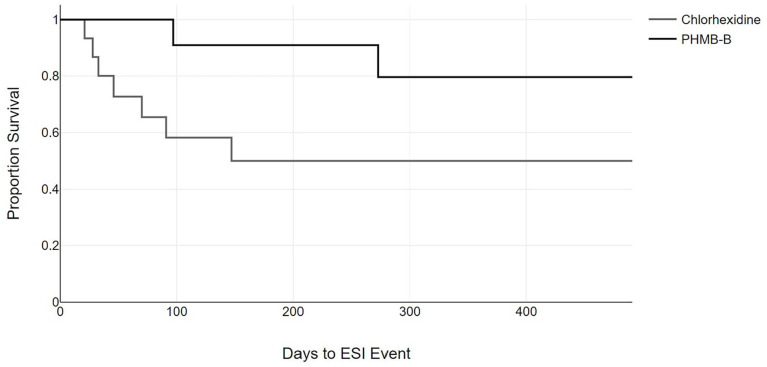
Period of time free of exit-site infections. The gray line represents patients who received the chlorhexidine treatment; the black line represents patients who received the polyhexanide treatment (log-rank 1.32; *p* = 0.250).

Finally, we analyzed the probability of developing a CRB and ESI event following initiation of hemodialysis using the Kaplan-Meier method, finding a significant difference in the probability of developing a CRB event between both groups (Figure 1). No differences were found in the probability of developing an ESI event between both groups (Figure 2).

## Discussion

Catheter-related infection pathogenesis is complex. The main sources of infection in tunneled catheters are catheter hub colonization, followed by peri-catheter skin colonization due to patient or healthcare professional manipulation.^14^ The hydrophobic interactions between the microorganism and the biosynthetic material of the catheter facilitate their adhesion and formation of the biofilm, an exopolysaccharide matrix that hinders the penetration of antimicrobials into the colony and its eradication, but allows the outflow of small amounts of bacteria to the bloodstream, causing bacteremia.^15^ Clinicians should always consider this phenomenon as a source of infection, despite being unable to monitor in vivo the presence of biofilms. PHMB-B has demonstrated high antibiofilm activity,^9^ which is why we decided to try this agent.

After the initiation of the PHMB-B protocol, our cohort showed a clear reduction in the CRB rate, and in the ESI rate, despite not being statistically significant. To our knowledge, this is the first study evaluating PHMB-B in the routine exit-site care of tunneled hemodialysis catheters. Despite being a small sample, our CRB rate with PHMB-B was 0.41 events/1000 catheter-days, which was remarkably low in comparison to the rate of 1.3 reported in larger series.^1^ The only CRB event after the protocol implementation occurred in an infant who had a severe complication (accidental catheter breakage, massive hemorrhage, and hypovolemic shock) several days after catheter placement, needing urgent and aggressive manipulation, suggesting that the routine exit-site care protocol would not have influenced the risk of infection in this patient. We hypothesize that the biofilm reduction in skin and catheter bacterial reservoirs due to the application of PHMB-B could be responsible for the reduction in the CRB rate. The longer effect duration of PHMB-B, up to 48 h after application, could also help minimize recolonization until next routine care.

These findings could be applicable not only in children, or tunneled catheters, but also in other types of vascular accesses where infections are also among their main complications.

## Limitations

Our study was mainly limited by its retrospective nature. Despite active vigilance and thorough filling of clinical charts and registries from our nurses and physicians regarding exit-site inspection and infectious events, there could have been other confounders we did not measure.

At the same time, our study has a small sample size. This was a single-center study, and end-stage chronic kidney disease in childhood had a low prevalence. However, our observations could act as a starting point for a larger, multicentric study.

Finally, because of the definition we chose, not all CRB/ESI events had a positive culture. Despite being aware that this issue can overestimate the number of events, in our opinion, our definition has more resemblance to the real costs derived from a CRB event in terms of diagnostic and therapeutic workup.

## Conclusion

Our study showed that the use of PHMB-B in routine exit-site care of hemodialysis tunneled catheters could reduce the rate of catheter-related bacteremia in our population. Despite the study limitations, these observations are promising, and could act as a starting point for a prospective, multicenter, randomized controlled trial. If these findings are proven true, the use of PHMB-B could be extrapolated to routine care of other types of vascular accesses.
